# Supplementary material for: Spotlight on the past: Focusing attention on long-term memory
Source: Mem Cognit. 2026 Mar 18;54(6):2031–61. doi: 10.3758/s13421-026-01854-w (PMC13461952; doi:10.3758/s13421-026-01854-w)
Supplement: Supplementary file 1 [file 13421_2026_1854_MOESM1_ESM.docx]

**Appendix A: Words Used in Experiment 7**

*Proportion of subjects identifying each spoken word correctly on the typing test, expressed as Levenshtein distance = 0 (LD = 0), and proportion of subjects identifying the word with no more than 2 typing errors (LD ≤ 2).*

| **Word** | **LD = 0** | **LD ≤ 2** |  | **Word** | **LD = 0** | **LD ≤ 2** |
| --- | --- | --- | --- | --- | --- | --- |
| BATCH | 0.8542 | 0.8958 |  | DRINK | 0.9375 | 1.0000 |
| BEARD | 0.9375 | 0.9583 |  | DRIVE | 1.0000 | 1.0000 |
| BEAST | 0.9792 | 1.0000 |  | DRONE | 0.8333 | 0.9792 |
| BEING | 0.9792 | 1.0000 |  | DROVE | 0.9583 | 0.9792 |
| BENCH | 0.9167 | 1.0000 |  | DROWN | 0.8750 | 0.9792 |
| BIRTH | 0.8333 | 1.0000 |  | DUTCH | 0.8542 | 0.9167 |
| BLACK | 0.9792 | 1.0000 |  | EARTH | 0.9583 | 0.9792 |
| BLADE | 0.9375 | 0.9375 |  | FAINT | 0.9167 | 1.0000 |
| BLAME | 0.9375 | 0.9792 |  | FAULT | 0.9167 | 0.9792 |
| BLANK | 0.7292 | 1.0000 |  | FILTH | 0.8333 | 0.9583 |
| BLAZE | 0.8333 | 0.9792 |  | FIRST | 0.9583 | 1.0000 |
| BLEAK | 0.7708 | 0.9792 |  | FLASH | 0.9167 | 1.0000 |
| BLIMP | 0.8333 | 1.0000 |  | FLECK | 0.7083 | 0.9583 |
| BLIND | 0.8958 | 0.9792 |  | FLESH | 0.8958 | 1.0000 |
| BLOCK | 0.8958 | 1.0000 |  | FLICK | 0.8958 | 1.0000 |
| BLUSH | 0.8542 | 0.9792 |  | FLING | 0.7917 | 0.8750 |
| BOAST | 0.8958 | 0.9792 |  | FLIRT | 0.8958 | 1.0000 |
| BOUND | 0.8958 | 0.9792 |  | FLOAT | 0.8958 | 1.0000 |
| BRACE | 0.9375 | 1.0000 |  | FLOWN | 0.9792 | 1.0000 |
| BRAID | 0.8125 | 0.9375 |  | FLUNG | 0.7708 | 0.9167 |
| BRAND | 0.9583 | 0.9792 |  | FLUSH | 0.5833 | 0.9792 |
| BRAVE | 0.8750 | 1.0000 |  | FORCE | 0.9167 | 1.0000 |
| BRIDE | 0.8542 | 0.9583 |  | FORGE | 0.8542 | 0.9792 |
| BRING | 0.8958 | 1.0000 |  | FOUND | 0.9792 | 1.0000 |
| BRISK | 0.8750 | 1.0000 |  | FRAIL | 0.8958 | 0.9792 |
| BROIL | 0.8750 | 0.9375 |  | FRAME | 0.9167 | 1.0000 |
| BROKE | 0.9375 | 1.0000 |  | FRAUD | 0.8958 | 0.9583 |
| BROWN | 0.9583 | 1.0000 |  | FREAK | 0.9583 | 1.0000 |
| BUDGE | 0.6667 | 0.8125 |  | FRESH | 0.8958 | 0.9792 |
| BUNCH | 0.8542 | 0.9375 |  | FRONT | 0.9167 | 0.9583 |
| BURNT | 0.8958 | 0.9792 |  | FROST | 0.8125 | 0.9792 |
| CAUSE | 0.8125 | 0.9583 |  | FROWN | 0.9167 | 0.9792 |
| CHAIN | 0.9583 | 1.0000 |  | FROZE | 0.8958 | 1.0000 |
| CHAMP | 0.8542 | 1.0000 |  | GHOST | 0.8750 | 0.9792 |
| CHANT | 0.9375 | 1.0000 |  | GLAND | 0.8125 | 1.0000 |
| CHARM | 0.9792 | 1.0000 |  | GLARE | 0.8333 | 0.9792 |
| CHEAT | 0.9167 | 0.9583 |  | GLIDE | 0.9167 | 1.0000 |
| CHEST | 0.8958 | 1.0000 |  | GLINT | 0.8333 | 0.9583 |
| CHILD | 0.9583 | 1.0000 |  | GLOAT | 0.8958 | 0.9792 |
| CHIME | 0.8542 | 0.9167 |  | GLOBE | 0.9375 | 1.0000 |
| CHUNK | 0.8958 | 0.9792 |  | GLOVE | 0.9375 | 1.0000 |
| CHURN | 0.8750 | 0.9375 |  | GRACE | 0.9583 | 1.0000 |
| CLANG | 0.6667 | 0.9583 |  | GRAND | 0.8333 | 1.0000 |
| CLASH | 0.9167 | 1.0000 |  | GRANT | 0.9167 | 1.0000 |
| CLEAN | 0.9792 | 0.9792 |  | GRAPE | 0.9375 | 0.9583 |
| CLEAR | 0.9583 | 0.9792 |  | GRASP | 0.8958 | 1.0000 |
| CLEFT | 0.8333 | 1.0000 |  | GRAVE | 0.9375 | 1.0000 |
| CLERK | 0.8125 | 0.9792 |  | GRIME | 0.8750 | 1.0000 |
| CLIMB | 0.9167 | 1.0000 |  | GRIPE | 0.8542 | 1.0000 |
| CLING | 0.8333 | 0.8750 |  | GROUP | 0.8958 | 1.0000 |
| CLINK | 0.8542 | 0.9792 |  | GROVE | 0.8958 | 0.9375 |
| CLOVE | 0.7083 | 1.0000 |  | GRUNT | 0.7708 | 0.9792 |
| CLOWN | 0.9167 | 1.0000 |  | GUIDE | 0.9792 | 1.0000 |
| COAST | 0.9375 | 1.0000 |  | GUILD | 0.7083 | 0.8958 |
| COUGH | 0.8542 | 0.9375 |  | GUILT | 0.8958 | 0.9792 |
| COULD | 0.9167 | 0.9583 |  | HAUNT | 0.7917 | 0.9583 |
| COUNT | 0.9167 | 1.0000 |  | HINGE | 0.7917 | 0.9792 |
| CRAFT | 0.9792 | 1.0000 |  | HOUSE | 0.9583 | 1.0000 |
| CRAMP | 0.8542 | 1.0000 |  | JOINT | 0.9167 | 1.0000 |
| CRASH | 0.8333 | 1.0000 |  | JUDGE | 0.9167 | 0.9583 |
| CRATE | 0.8333 | 1.0000 |  | KNIFE | 0.8958 | 1.0000 |
| CRAVE | 0.9167 | 0.9583 |  | LARGE | 0.9792 | 1.0000 |
| CRAZE | 0.8750 | 0.9375 |  | LATCH | 0.8333 | 0.8750 |
| CREAM | 0.9583 | 0.9792 |  | LAUGH | 0.9375 | 0.9792 |
| CREPT | 0.7500 | 1.0000 |  | LEARN | 0.9375 | 1.0000 |
| CREST | 0.9167 | 1.0000 |  | LEASH | 0.8333 | 0.9583 |
| CRIED | 0.8333 | 0.8750 |  | LEAST | 0.8750 | 1.0000 |
| CRIME | 0.9167 | 1.0000 |  | LOVED | 0.9583 | 1.0000 |
| CRISP | 0.9583 | 0.9792 |  | LUNCH | 0.8542 | 0.9583 |
| CRUDE | 0.7917 | 0.8542 |  | LUNGE | 0.6042 | 0.9167 |
| CRUST | 0.8958 | 0.9792 |  | MARCH | 0.8333 | 0.9792 |
| DANCE | 1.0000 | 1.0000 |  | MARSH | 0.7500 | 0.9792 |
| DEPTH | 0.8125 | 0.9375 |  | MATCH | 0.8750 | 0.9583 |
| DITCH | 0.8750 | 0.9792 |  | MONTH | 0.8958 | 0.9792 |
| DOUBT | 0.8958 | 1.0000 |  | MOUND | 0.8333 | 0.9583 |
| DRAFT | 0.8750 | 1.0000 |  | MOURN | 0.9167 | 0.9792 |
| DRAPE | 0.8125 | 0.9583 |  | MOUSE | 0.9375 | 0.9583 |
| DRAWN | 0.8542 | 0.9583 |  | MOUTH | 0.8750 | 0.9583 |
| DREAM | 0.9792 | 0.9792 |  | MULCH | 0.7917 | 0.9583 |
| DRIFT | 0.9792 | 0.9792 |  | NOISE | 0.9583 | 1.0000 |

| **Word** | **LD = 0** | **LD ≤ 2** |  | **Word** | **LD = 0** | **LD ≤ 2** |
| --- | --- | --- | --- | --- | --- | --- |
| NORTH | 0.9583 | 1.0000 |  | SNAIL | 0.9792 | 1.0000 |
| NURSE | 0.9375 | 0.9792 |  | SNAKE | 0.9583 | 1.0000 |
| OUNCE | 0.8333 | 0.9167 |  | SNARE | 0.8333 | 0.9583 |
| PAINT | 0.9167 | 0.9792 |  | SNARL | 0.7292 | 1.0000 |
| PANTS | 0.8958 | 0.9792 |  | SNORE | 0.9375 | 1.0000 |
| PEACH | 0.8958 | 0.9792 |  | SOLVE | 0.9167 | 0.9792 |
| PITCH | 0.8750 | 0.9792 |  | SOUND | 0.9792 | 1.0000 |
| PLACE | 1.0000 | 1.0000 |  | SOUTH | 0.8333 | 0.8958 |
| PLANK | 0.8542 | 0.9792 |  | SPACE | 0.8958 | 0.9792 |
| PLANT | 0.9375 | 1.0000 |  | SPARE | 0.9375 | 1.0000 |
| PLATE | 0.9583 | 1.0000 |  | SPARK | 0.9583 | 1.0000 |
| PLUCK | 0.8750 | 0.9792 |  | SPEND | 0.9583 | 1.0000 |
| PLUSH | 0.8750 | 0.9583 |  | SPENT | 0.9375 | 1.0000 |
| POINT | 0.9583 | 1.0000 |  | SPICE | 0.9792 | 1.0000 |
| POISE | 0.8750 | 1.0000 |  | SPIKE | 0.9583 | 1.0000 |
| PORCH | 0.8750 | 1.0000 |  | SPINE | 0.9583 | 1.0000 |
| PRICE | 0.8542 | 1.0000 |  | SPORE | 0.8958 | 1.0000 |
| PRIDE | 0.9375 | 1.0000 |  | SPORT | 0.9375 | 1.0000 |
| PRIME | 0.9583 | 1.0000 |  | SPRAY | 0.9583 | 1.0000 |
| PRINT | 0.9583 | 1.0000 |  | SQUAT | 0.8333 | 0.9792 |
| PROBE | 0.9583 | 1.0000 |  | SQUID | 0.8542 | 0.9792 |
| PRONE | 0.8958 | 1.0000 |  | STACK | 0.7708 | 1.0000 |
| PROUD | 0.8542 | 1.0000 |  | STAGE | 0.9375 | 0.9792 |
| PROVE | 0.8125 | 1.0000 |  | STAIN | 0.9167 | 0.9583 |
| PUNCH | 0.9583 | 1.0000 |  | STALE | 0.6875 | 0.9792 |
| PURSE | 0.8542 | 1.0000 |  | STAMP | 0.9792 | 1.0000 |
| QUEST | 0.9375 | 0.9792 |  | STAND | 1.0000 | 1.0000 |
| QUICK | 0.9583 | 0.9792 |  | STARK | 0.7708 | 1.0000 |
| QUILT | 0.8542 | 0.9375 |  | STEAD | 0.8542 | 1.0000 |
| QUITE | 0.9167 | 1.0000 |  | STEAM | 0.9375 | 0.9583 |
| RAISE | 0.8333 | 0.9792 |  | STICK | 0.8958 | 1.0000 |
| RANCH | 0.8542 | 0.9792 |  | STINK | 0.8958 | 0.9792 |
| REACH | 0.7917 | 0.9583 |  | STOKE | 0.9375 | 1.0000 |
| REALM | 0.7917 | 0.8958 |  | STOLE | 0.9167 | 1.0000 |
| RIDGE | 0.7917 | 0.8750 |  | STONE | 0.9375 | 0.9792 |
| ROAST | 0.9583 | 1.0000 |  | STORE | 0.9375 | 0.9792 |
| ROUGH | 0.8958 | 0.9583 |  | STORM | 0.9583 | 1.0000 |
| ROUND | 0.9375 | 1.0000 |  | STOVE | 0.8958 | 1.0000 |
| SAUCE | 0.8333 | 0.9375 |  | STRAP | 0.8333 | 0.9792 |
| SCALE | 0.8333 | 0.9167 |  | STRAW | 0.7917 | 0.9167 |
| SCARE | 0.9583 | 0.9792 |  | STRAY | 0.8542 | 0.9792 |
| SCOPE | 0.9583 | 1.0000 |  | STREW | 0.7708 | 0.8958 |
| SCORE | 0.9375 | 0.9792 |  | STRIP | 0.9167 | 0.9792 |
| SCRAP | 0.8750 | 0.9375 |  | STUCK | 0.9375 | 0.9792 |
| SCREW | 0.9167 | 0.9583 |  | STUNG | 0.7500 | 0.9792 |
| SCRUB | 0.9167 | 0.9792 |  | SWAMP | 0.9375 | 1.0000 |
| SHADE | 0.9375 | 1.0000 |  | SWEAT | 0.7083 | 1.0000 |
| SHAKE | 0.9583 | 1.0000 |  | SWIFT | 0.9583 | 1.0000 |
| SHAME | 0.9583 | 1.0000 |  | SWING | 0.9375 | 0.9792 |
| SHAPE | 0.9583 | 1.0000 |  | SWIPE | 0.8958 | 1.0000 |
| SHARE | 0.8958 | 0.9375 |  | SWIRL | 0.8333 | 0.9583 |
| SHARK | 0.9792 | 1.0000 |  | SWORD | 0.8958 | 0.9583 |
| SHARP | 0.9583 | 0.9792 |  | TEACH | 0.9583 | 0.9792 |
| SHAVE | 0.9792 | 1.0000 |  | THINK | 0.9167 | 0.9792 |
| SHELF | 0.9167 | 0.9583 |  | THIRD | 0.8958 | 0.9792 |
| SHIFT | 1.0000 | 1.0000 |  | THROW | 0.8750 | 0.9167 |
| SHINE | 0.9792 | 1.0000 |  | THUMP | 0.7500 | 0.9583 |
| SHOCK | 0.7917 | 0.9792 |  | TORCH | 0.8542 | 1.0000 |
| SHORT | 0.9583 | 0.9792 |  | TOUCH | 0.9167 | 0.9792 |
| SHOUT | 0.9375 | 1.0000 |  | TRACE | 0.9375 | 0.9792 |
| SHOVE | 0.8750 | 0.9792 |  | TRACK | 0.8542 | 1.0000 |
| SKIRT | 0.8958 | 0.9375 |  | TRAIL | 0.9167 | 0.9583 |
| SLACK | 0.8333 | 0.9375 |  | TRAIN | 1.0000 | 1.0000 |
| SLANG | 0.7500 | 0.9375 |  | TRASH | 0.9167 | 1.0000 |
| SLANT | 0.8333 | 0.9792 |  | TRIBE | 0.8542 | 1.0000 |
| SLATE | 0.9375 | 1.0000 |  | TRICK | 0.8958 | 1.0000 |
| SLEPT | 0.8750 | 0.9583 |  | TRIED | 0.7292 | 0.9375 |
| SLICK | 0.7292 | 0.9583 |  | TRUCK | 0.9375 | 1.0000 |
| SLIDE | 0.8750 | 0.8958 |  | TRUNK | 0.9792 | 1.0000 |
| SLIME | 0.8750 | 0.9792 |  | TWINE | 0.9583 | 1.0000 |
| SLOPE | 0.9375 | 1.0000 |  | TWIRL | 0.7708 | 0.8958 |
| SLOTH | 0.7708 | 0.9375 |  | VOICE | 0.9375 | 0.9792 |
| SLUMP | 0.7292 | 1.0000 |  | WHALE | 0.9167 | 0.9792 |
| SMACK | 0.8333 | 1.0000 |  | WHILE | 0.8542 | 0.9792 |
| SMART | 0.9583 | 1.0000 |  | WIDTH | 0.6875 | 0.9375 |
| SMELT | 0.8542 | 0.9792 |  | WOUND | 0.8958 | 0.9792 |
| SMILE | 0.9792 | 1.0000 |  | WRATH | 0.6042 | 0.7917 |
| SMIRK | 0.6875 | 0.9375 |  | WRONG | 0.9375 | 0.9583 |
| SMOKE | 0.9583 | 1.0000 |  | YEARN | 0.7917 | 0.9375 |
| SNACK | 0.9375 | 1.0000 |  | YOUNG | 0.9792 | 1.0000 |

**Appendix B: Picture Names Used in Experiment 8**

*Proportion of subjects identifying each picture correctly on the typing test, expressed as Levenshtein distance = 0 (LD = 0), and proportion of subjects identifying the word with no more than 2 typing errors (LD ≤ 2).*

| **Word** | **LD = 0** | **LD < 2** |  | **Word** | **LD = 0** | **LD < 2** |
| --- | --- | --- | --- | --- | --- | --- |
| BELT | 0.8438 | 0.9688 |  | CAMEL | 0.9063 | 1.0000 |
| BOWL | 0.8438 | 1.0000 |  | CHAIN | 0.9688 | 0.9688 |
| COMB | 0.9688 | 1.0000 |  | CHAIR | 0.7813 | 0.9688 |
| DESK | 0.9063 | 0.9688 |  | CIGAR | 0.5938 | 0.6250 |
| DRUM | 0.8750 | 0.9375 |  | CLOUD | 0.8750 | 0.9375 |
| DUCK | 0.9375 | 1.0000 |  | CLOWN | 0.8750 | 0.9688 |
| FISH | 0.9688 | 1.0000 |  | CROWN | 0.8750 | 1.0000 |
| FLAG | 0.9063 | 1.0000 |  | GLOVE | 0.9375 | 0.9688 |
| FORK | 0.8750 | 0.9375 |  | HEART | 0.9688 | 1.0000 |
| FROG | 1.0000 | 1.0000 |  | HORSE | 0.9688 | 1.0000 |
| HAIR | 0.0000 | 0.0000 |  | HOUSE | 0.9063 | 0.9688 |
| HAND | 0.9688 | 1.0000 |  | KNIFE | 1.0000 | 1.0000 |
| IRON | 1.0000 | 1.0000 |  | LEMON | 0.8438 | 1.0000 |
| LEAF | 0.9688 | 0.9688 |  | SCREW | 0.8438 | 0.9063 |
| LION | 0.8438 | 0.9375 |  | SHIRT | 0.9063 | 0.9688 |
| NAIL | 0.8750 | 0.9063 |  | SKIRT | 0.8125 | 0.8750 |
| NOSE | 0.9688 | 1.0000 |  | SNAKE | 0.9375 | 0.9375 |
| PEAR | 0.9375 | 0.9688 |  | SWING | 0.8125 | 0.8438 |
| RING | 0.9063 | 1.0000 |  | TABLE | 0.8438 | 0.9375 |
| SLED | 0.7500 | 0.8125 |  | THUMB | 0.8438 | 0.8750 |
| SOCK | 0.9375 | 0.9375 |  | TIGER | 0.9063 | 0.9375 |
| STAR | 0.8125 | 0.9688 |  | TRUCK | 0.8750 | 0.9688 |
| VASE | 0.8438 | 0.8750 |  | WATCH | 1.0000 | 1.0000 |
| VEST | 0.8438 | 0.8750 |  | ZEBRA | 0.9375 | 0.9688 |
